# Response to Comment on: “The Effect of Preterm Birth on Maximal Aerobic Exercise Capacity and Lung Function in Healthy Adults: A Systematic Review and Meta-Analysis”

**DOI:** 10.1007/s40279-023-01810-7

**Published:** 2023-01-31

**Authors:** Thomas Gostelow, Eric J. Stöhr

**Affiliations:** 1 School of Sport and Health Sciences, Cardiff Metropolitan University, Cardiff, UK; 2 Department of Medicine, Division of Cardiology, Columbia University Irving Medical Center, New York City, NY USA; 3 COR-HELIX (CardiOvascular Regulation and Exercise Laboratory – Integration and Xploration), Institute of Sport Science, Leibniz University Hannover, Am Moritwinkel 6, 30167 Hannover, Germany

Dear Editor,

We have received the comments by Manferdelli et al. [1] on our recently published article [2] and gladly share our response in the journal. The discussion points raised by the authors are of great importance in relation to the broader topic. However, we are surprised by the specific points raised by Manferdelli et al. [1] as we consider them transparently addressed in our original investigation [2]. Below, we have attempted to clarify these points accordingly.

## $\dot{V}{\text{O}}_{\text{2peak}}$ and Pulmonary Function are Not Always Reduced: Indeed, But the Pooled Data Clearly Suggest a Lower Function

With regard to the first and second point, we concur that individual studies on peak oxygen uptake ($\dot{V}{\text{O}}_{\text{2peak}}$) and lung function have provided mixed results. In fact, this was one of the main reasons for conducting our more focused meta-analysis following the systematic review. From our Table 1, it is evident that several of the previous studies did not find a statistically supported “difference”. Importantly, the majority of the studies included in the systematic review did not report physical activity, a key confounding factor. For the few studies that did report physical activity, we showed in our Figs. 2 and 3 that there was also mixed evidence (six out of 11 studies for $\dot{V}{\text{O}}_{\text{2peak}}$ and three out of six studies for forced expiratory volume in 1 s indicated ‘no effect’ of premature birth). Consequently, we highlighted that the current evidence—based upon individual studies alone—was not sufficient to determine whether premature birth has a negative impact on $\dot{V}{\text{O}}_{\text{2peak}}$ and pulmonary function in adults: “Although the majority of previous research implies that being born preterm has a negative impact on lung function and exercise capacity, there is sufficient alternative evidence to suggest that preterm birth may not be a clear factor for reduced $\dot{V}{\text{O}}_{\text{2max}}$” [2].

In addition, Manferdelli et al. [1] suggest that we have omitted several articles from our systematic review. For the following reasons, we do not think that this criticism is warranted. Of the five references referred to by the authors, one article was already included in our systematic review for both $\dot{V}{\text{O}}_{\text{2peak}}$ and forced expiratory volume in 1 s [3], one article was not eligible according to our transparently stated inclusion period [4], one article included children only and was therefore not eligible as per our explicit focus on adults [5], and another article did not appear in the original search, which may be owing to differing pre-defined key terms [6]. The fifth article by Debevec et al. [7] could have been included in our systematic review and we apologise for the oversight. However, the article by Debevec et al. [7] and all (!) of the other original articles eligible for inclusion in the meta-analysis reported lower mean values for $\dot{V}{\text{O}}_{\text{2peak}}$ and pulmonary function in the prematurely born adults compared with healthy controls [2]. Therefore, we are confident in our conclusion that the empirical evidence from the pooled data improves our current understanding and strongly indicates a reduced $\dot{V}{\text{O}}_{\text{2peak}}$ and pulmonary function in adults born pre-term.

## BPD is an Important Factor—But Not in Relation to Our Research Question

The third point raised by Manferdelli et al. [1] surprises us because we have explicitly stated in our article that broncho-pulmonary dysplasia (BPD) is important (see the extensive part of the introduction in [2]), but that we also had clear reasons for not including individuals with BPD in our article because they are almost too obviously predestined to have a lower exercise capacity (“Accordingly, adults who were born preterm with BPD are already more likely to have a reduced cardiorespiratory fitness …. In contrast, there are numerous individuals born prematurely who do not have BPD, but whether they have a reduced exercise capacity is not clear” [2]). Of course, future studies are warranted to capture aspects that our analysis did not aim to examine. However, the purpose of our focused analysis was to gain insight from a homogeneous study sample and avoid major confounding factors such as BPD and physical activity patterns. We think that the results based upon such an approach significantly extend the current discussion in the literature because there seems to exist a negative effect of premature birth beyond some of the more obvious (confounding) factors. Consequently, here, we are pleased to be able to confirm our previous conclusion: “in adults born preterm, exercise interventions may result in a lower benefit compared with adults born at term, and additional therapeutic interventions or higher exercise intensities/volumes may be required.”
